# MiRNA-153 attenuates progression of non-small cell lung cancer through targeting positive regulatory/SET domain 2

**DOI:** 10.4314/ahs.v24i3.24

**Published:** 2024-09

**Authors:** Ji Chen, Shiliang Xie, Miao Feng, Dan Wang

**Affiliations:** 1 Department of Thoracic Surgery, Huashan Hospital Affiliated to Fudan University, Shanghai, China; 2 Department of Cardiothoracic Surgery, Shanghai Tongji hospital/Tongji hospital Affiliated to Tongji University, Shanghai, China; 3 Department of Traditional Chinese Medicine, Changzheng Hospital, Naval Medical University, Shanghai, China

**Keywords:** Non-small cell lung cancer, miR-153, positive regulatory/SET domain 2, Janus kinase-signal transducer and activator of transcription (JAK/STAT) signaling pathway

## Abstract

**Background:**

To explore whether micro ribonucleic acid (miR)-153 regulates positive regulatory/SET domain 2 (PRDM2) in a targeted manner and affects the proliferation and apoptosis of non-small cell lung cancer (NSCLC) A549 cells.

**Methodology:**

The expressions of miR-153 and PRDM2 in NSCLC tissues and A549 cells were detected by quantitative real-time polymerase chain reaction (qRT-PCR). TargetScan was utilized to predict miR-153 target genes. Methyl thiazolyl tetrazolium (MTT) and terminal deoxynucleotidyl transferase dUTP nick end labeling (TUNEL) assays were carried out to study the cell proliferation and apoptosis. Western blotting was performed to examine the changes in the proteins in the Janus kinase-signal transducer and activator of transcription (JAK/STAT) signaling pathway following the miR-153 overexpression.

**Results:**

The expression of miR-153 was decreased and that of PRDM2 was increased in NSCLC tissues and cells. Target genes regulated by miR-153 participated in self-vascular development, miRNA metabolic process and the Wnt signaling pathway. The overexpression of miR-153 led to an obvious reduction in the proliferation ability of A549 cells, a notable increase in apoptotic cells, and significant decreases in phosphorylated (p)-JAK2 and p-STAT3 proteins. Dual-luciferase reporter gene assay revealed that miR-153 could directly modulate the expression level of PRDM2.

**Conclusion:**

MiR-153 directly targets PRDM2 and affects A549 cell proliferation and apoptosis through the JAK/STAT pathway.

## Introduction

Lung cancer is the major cause of cancer-related deaths globally, accounting for 18.4% of the total number of cancer-related deaths, and there are about 2.5 million new cases and 1.5 million deaths each year^1^,^2^. Lung cancer can be divided into two main histological types, namely, small cell lung cancer (SCLC) and non-small cell lung cancer (NSCLC), and the latter is the most common. NSCLC mainly has two pathological subtypes, namely, adenocarcinoma (ADC) and squamous cell carcinoma (SCC)^3^. Most NSCLC patients are diagnosed with the disease in the advanced stage, and the overall five-year survival rate is only 14-17% owing to the low detection rate of early lung cancer and poor therapeutic effect of advanced lung cancer^4^. Therefore, it is very necessary to fully understand the molecular and cytological processes of NSCLC and to detect NSCLC early for reducing the mortality rate of the disease^5^.

Over 90% of the genes in the mammalian genome are transcribed positively, but only a relatively small fraction of genes are responsible for translation, and a large proportion of genes participate in the synthesis of regulatory factors, including non-coding ribonucleic acids (ncRNAs)^6^,^7^. Micro RNAs (miRNAs), short single-stranded ncRNAs (with a length of about 22 nucleotides), bind to complementary sites in target messenger RNAs (mRNAs) on RNA-induced silencing complexes (RISCs) and induce mRNA degradation, so as to negatively modulate gene expression or inhibit translation^8^. In the past few decades, extensive research has been conducted for the biogenesis and biological effects of miRNAs^9^. Previous studies have manifested that miRNAs exert vital effects in the development of mammals, and their dysregulation can lead to diverse human diseases, including malignant tumors^10^. Therefore, miRNAs are widely involved in several events of cancer progression, such as proliferation, metastasis, chemical resistance, and epithelial-mesenchymal transition (EMT)^11^-^14^. According to the inhibition of different target genes, miRNAs can act as carcinogenic or cancer suppressor genes, and the functions of some miRNAs in cancer inhibition and promotion are controversial^15^,^16^. Dysregulation of miR-153 has recently been observed in multiple common human cancers, and it acts as a carcinogenic gene^17^,^18^ or a cancer suppressor gene^19^,^20^ in different types of cancers. It has been determined by previous studies that the miR-153 expression is significantly reduced in NSCLC tissues^21^, and influences the migration and invasion of human NSCLC by targeting ADAM192^22^.

MiR-153 is a miRNA that has been found to play a role in cancer. Its expression has been reported to be downregulated in several types of human cancers, including NSCLC^23^,^24^. The functions of miR-153 in cancer have been found to be context-dependent, with some studies reporting it as a tumor suppressor while others suggest it may act as an oncogene. For example, miR-153 has been shown to suppress the proliferation and invasion of hepatocellular carcinoma cells, while promoting apoptosis in colorectal cancer cells. Conversely, miR-153 has also been found to promote the proliferation and invasion of cervical cancer cells. In NSCLC, miR-153 has been shown to be significantly downregulated in tumor tissues compared to adjacent non-tumor tissues, and its expression has been shown to correlate with poor prognosis. Previous studies have also suggested that miR-153 may be involved in regulating NSCLC cell migration and invasion by targeting ADAM19. However, the specific molecular mechanisms underlying the role of miR-153 in NSCLC remain largely unknown^25^.

Positive regulatory/SET domain 2 (PRDM2) belongs to the PRDM gene family, which is a subfamily of Kruppel-like zinc finger gene products, with 19 members currently in human beings^26^,^27^. Located at chromosome 1p36 that is easily modified by deletion and modification, PRDM2 plays a variety of roles in numerous human malignant tumors, such as neuroblastoma^28^, hepatocellular tumor^29^, colorectal cancer^30^, ovary cancer^31^ and breast cancer^32^. As is known to all, the Janus activated kinase (JAK) signaling pathway and the signal transduction and transcriptional activator (STAT) signaling pathway exert crucial effects in modulating cell apoptosis, embryonic development, liver regeneration, glycolysis, inflammatory responses, EMT and angiogenesis^33^. Continuously activating JAK/STAT in diverse tumor cells can facilitate the malignant transformation of tumors^34^. In NSCLC, the phosphorylation of STAT activated by JAK takes up 22-65%^35^,^36^, so the JAK/STAT signaling pathway is the pivotal regulator of NSCLC^37^. Although miR-153 has been confirmed to be related to NSCLC, there are no reports indicating whether miR-153 participates in the pathogenesis of NSCLC through the JAK/STAT signaling pathway.

This study aims to explore the expression level of miR-153 in human NSCLC tissues and cells as well as the effect and mechanism of miR-153 on A549 cell proliferation and apoptosis through the JAK/STAT signaling pathway.

## Patients and methods

### Human NSCLC tissue samples

A total of 65 patients definitely diagnosed with NSCLC in our hospital were collected. Immediately after surgical resection, NSCLC tissue samples and adjacent normal tissue (control tissue) samples were obtained from patients and then stored in a refrigerator at −80°C for subsequent experiments. The inclusion criteria for this study were patients with a definite diagnosis of NSCLC, confirmed by pathological examination, and who underwent surgical resection in our hospital. Patients who provided written informed consent were also included. The exclusion criteria were patients who received chemotherapy or radiotherapy before surgery, patients with other malignancies or severe comorbidities, and patients who refused to participate in the study. Additionally, patients who had incomplete medical records or tissue specimens that were unsuitable for analysis were also excluded. This study was approved by the Ethics Committee of our hospital and the written informed consent was obtained from all patients.

### Target gene prediction and analysis

Target gene prediction is a crucial step in miRNA research to understand their biological functions. In this study, the online tool TargetScan was employed to predict the target genes of miR-153. TargetScan is a widely used algorithm that predicts the potential target genes of miRNAs based on sequence complementarity between the miRNA seed region and the 3′UTR of the mRNA. The predicted target genes were then analysed using Metascape, a comprehensive web-based tool for gene annotation and analysis. Metascape provides a variety of functional enrichment and pathway analysis tools to interpret large-scale gene lists, including Gene Ontology (GO) analysis, Kyoto Encyclopedia of Genes and Genomes (KEGG) pathway analysis, and protein-protein interaction (PPI) network analysis. By analysing the predicted target genes of miR-153 using Metascape, we aimed to gain insight into the potential biological processes and pathways in which miR-153 may be involved.

### Cell culture

Human normal gastric mucosa BEAS-2B cell line and human NSCLC A549 cell line were purchased from Shanghai Institute of Biochemistry and Cell Biology, Chinese Academy of Sciences (Shanghai, China) and cultured in dulbecco's modified eagle medium (DMEM) (Invitrogen, Carlsbad, CA, USA) containing 10% fetal bovine serum (FBS) (Gibco, Rockville, MD, USA) and 1% penicillin/streptomycin solution in an incubator with 5% CO_2_ at 37°C.

### Cell transfection

In this study, A549 cells were selected as the cell model, which were maintained in RPMI-1640 medium supplemented with 10% fetal bovine serum (FBS) and 1% penicillin/streptomycin in a humidified atmosphere with 5% CO2 at 37°C. Before transfection, the cells were seeded in 24-well plates at a density of 5×10^4 cells per well and allowed to adhere for 24 h. The miRNA transfection reagent was used according to the manufacturer's instructions. Briefly, 100 nM of miR-153 mimic and miRNC were diluted in Opti-MEM medium, and then added to the cells along with the transfection reagent. After 24 h of transfection, total RNAs were extracted from the collected cells using TRIzol reagent following the manufacturer's protocol. The expression level of miR-153 was measured by quantitative real-time PCR (qRT-PCR). Additionally, 48 h after transfection, total proteins were extracted from the collected cells using RIPA buffer for subsequent analysis of protein expression by western blot.

### Quantitative real-time polymerase chain reaction (qRT-PCR)

TRIzol reagent was utilized to extract total RNAs. Complementary deoxyribose nucleic acid (cDNA) synthesis was performed using the TaqMan MicroRNA Array kit (Applied Biosystems, Foster City, CA, USA). Then qRTPCR was performed using primers. U6 was used as an endogenous control to standardize the expression of miR-153, while GAPDH was used as an internal reference to standardize the expression of PRDM2. Finally, all relative expression levels were measured by the 2-ΔΔCt method. Primers were shown in Table 1.

**Table 1:** Primer sequences

Index	Forward primer sequence (5′-3′)	Reverse primer sequence (5′-3′)
GAPDH	GGAGCGAGATCCCTCCAAAAT	GGCTGTTGTCATACTTCTCATGG
U6	CCCTTCGGGGACATCCGATA	TTTGTGCGTGTCATCATTTG
MiR-153	ACACTCCAGCTGGGTTGCATAGTCACAAA	CAGTGCGTGTCGCGTGGAGT
PRDM2	CCTCAAGTAGGTTTAAGAGGCG	GGTGAGTCGCTGTGACTTTCTA

### Immunohistochemistry (IHC)

SCLC tumor tissues were fixed in 10% formalin buffer and treated using a paraffin tissue processor. Then the tissue sections were subjected to IHC to evaluate the expression of PRDM2 in tumor tissues. After paraffin removal and fluid replacement, the sections were incubated in 3% H2O2 to block endogenous peroxidase in them and washed by phosphate-buffered saline (PBS) containing 0.05 mol/L EDTA (ethylenediaminetetraacetic acid) and by 4% paraformaldehyde in sequence. After that, the tissues were sealed in a blocking buffer for 1 h. Next, in the presence of 10% rabbit serum, 5 µm sections were incubated with anti-PRDM2 antibody (Santa Cruz, Santa Cruz, CA, USA) at room temperature overnight. Following washing, the sections were incubated with horse radish peroxidase (HRP)-conjugated IgG secondary antibody for 1 h. At last, glass slides were counterstained with hematoxylin to stain nuclei and examined under an optical microscope (Olympus, Tokyo, Japan).

### Methyl thiazolyl tetrazolium (MTT) assay

Cell proliferation was estimated by MTT (Sigma-Aldrich, St. Louis, MO, USA) according to the manufacturer's scheme. Briefly, A549 cells were inoculated into 96-well plates, and each group of cells was incubated with MTT solution (5 mg/mL) diluted with 50 µL of PBS at 37°C for 4 h on the 1^st^, 2^nd^, 3^rd^, 4^th^ and 5^th^ day after transfection, respectively. After the removal of the cell supernatant, the reaction was terminated by adding 200 µL of dimethyl sulfoxide (DMSO) (Sigma-Aldrich, St. Louis, MO, USA). Ultimately, the optical density at 595 nm (OD_595_) was read using a microplate reader (Bio-Rad, Hercules, CA, USA).

### Terminal deoxynucleotidyl transferase dUTP nick end labeling (TUNEL) assay

A549 cells were fixed with formaldehyde and washed by PBS for 3 times. Thereafter, the cells were permeabilized in 1% Tritonx-100 at room temperature and reacted with TdT reaction solution, followed by color development. Lastly, A549 cells were observed under the microscope, and TUNEL^+^ cells were counted (https://www.biolifesas.org/EN/home).

### Western blotting analysis of proteins

Radioimmunoprecipitation assay (RIPA) lysis buffer (Beyotime, Shanghai, China) was utilized to extract cell proteins. The same quantity of proteins (30 µg) were subjected to 10% sodium dodecyl sulphate-polyacrylamide gel electrophoresis (SDS-PAGE) and then transferred onto a polyvinylidene fluoride (PVDF) membrane (Millipore, Billerica, MA, USA). The membrane was sealed with 5% skim milk diluted with tris buffered saline-tween (TBST) for 1 h and incubated with antibodies against JAK2 (1:1000), phosphorylated (p)-JAK2 (1:2000), STAT3 (1:1000) and p-STAT3 (1:2000) at 4°C. The next day, the membrane was incubated with HRP-conjugated secondary antibody (1:2000). At last, enhanced chemiluminescence (ECL)-Plus detection reagent was applied to detect protein expression signals, with β-actin as an internal control.

### Statistical analysis

The statistical analysis was performed using IBM SPSS 22.0 software (IBM, Armonk, NY, USA). Continuous variables were presented as mean ± standard deviation (SD). The comparison of continuous variables between two groups was performed using the independent two-sample t-test. Additionally, a correlation analysis was performed to determine the relationship between miR-153 and PRDM2 expression. Furthermore, a dual-luciferase reporter gene assay was conducted to verify the regulatory relationship between miR-153 and PRDM2. All experiments were performed in triplicate, and the results were presented as mean ± SD. A p-value less than 0.05 was considered statistically significant.

## Results

### MiR-153 expression was reduced in NSCLC tissues and cells

**Figure 1:**
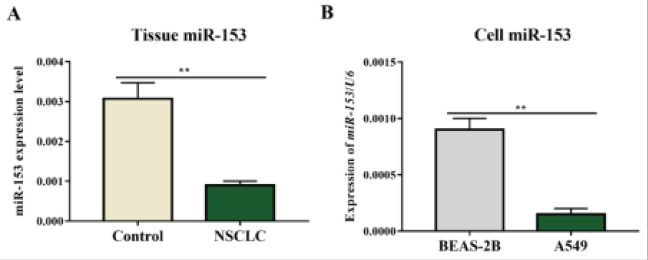
QRT-PCR revealed that the miR-153 expression was reduced in NSCLC tissues and cells. (A) Differential expression of miR-153 in NSCLC tissues and control tissues. (B) Differential expression of miR-153 in A549 cells and BEAS-2B cells. Note: Compared with that in control tissues, the expression of miR-153 in NSCLC tissues was decreased (**P<0.01). In comparison with that in BEAS-2B cells, the expression of miR-153 in A549 cells was decreased (**P<0.01)

MiR-153 expression levels were measured using quantitative reverse transcription PCR (qRT-PCR) analysis. For the comparison of miR-153 expression in NSCLC tissues and control tissues, a paired t-test was used, and the results showed a significant decrease in miR-153 expression in NSCLC tissues compared to control tissues (P<0.01, Figure 1A). For the analysis of miR-153 expression in A549 cells, an unpaired t-test was used, and the results showed a significant decrease in miR-153 expression in A549 cells compared to the negative control (P<0.01, Figure 1B). These results suggest that miR-153 expression is downregulated in NSCLC tissues and cells, which may indicate its potential role in the development and progression of NSCLC.

### Expression level of miR-153 and prognosis of NSCLC

**Figure 2:**
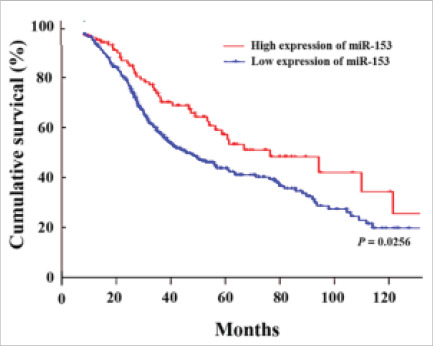
Kaplan-Meier survival analysis of miR-53 expression in NSCLC patients

The Kaplan-Meier survival analysis was performed using the log-rank test to determine the significance of the difference between the survival curves of NSCLC patients with low and high miR-153 expression. The overall survival rate was calculated and plotted against the follow-up time. The statistical significance was set at P<0.05. The survival analysis was performed using SPSS software version 23.0. The results showed that the difference between the survival curves was statistically significant (P=0.0256), indicating that miR-153 may have a prognostic value in NSCLC. The hazard ratio (HR) was also calculated to assess the risk of death in patients with low miR-153 expression compared to those with high miR-153 expression (Figure 2).

### MiR-153 target gene prediction and enrichment analysis

**Figure 3:**
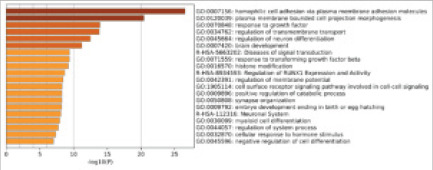
Statistics of GO entries enriched with miR-153 target genes. Note: Abscissa: -1og10 (P), i.e., P=the negative value of the logarithm of 10

TargetScan was utilized to predict miR-153 target genes, and 1886 target genes were obtained. PRDM2 was in the target gene list of miR-153. Then the target gene set of miR-153 was uploaded to the online tool Metascape for enrichment analysis. A series of biological processes and pathways involving target genes were obtained, which covered many categories, including histone modification, transmembrane transport and membrane protein regulation (Figure 3).

### PRDM2 expression was raised in NSCLC tissues and cells

**Figure 4:**
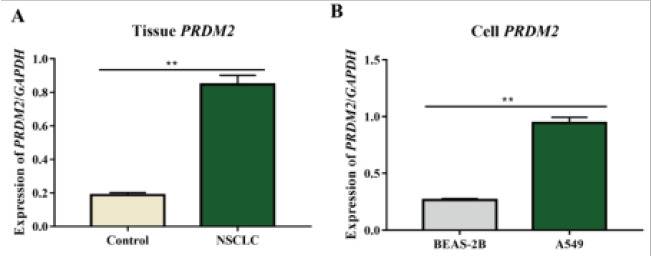
PRDM2 mRNA expression was raised in NSCLC tissues and A549 cells. Note: PRDM2 expression in NSCLC tissues (A) and A549 cells (B) was markedly increased compared with that in control tissues and BEAS-2B cells (**P<0.01)

In order to identify the PRDM2 expression in NSCLC tissues and cells, qRT-PCR and IHC were performed. QRT-PCR results manifested that the mRNA expression of PRDM2 was increased in NSCLC tissues (P<0.01, Figure 4A) and A549 cells (P<0.01, Figure 4B). According to IHC results, the protein expression of PRDM2 rose in NSCLC tissues (P<0.05).

### Changes in the proliferation and apoptosis of A549 cells after overexpression of miR-153

**Figure 5:**
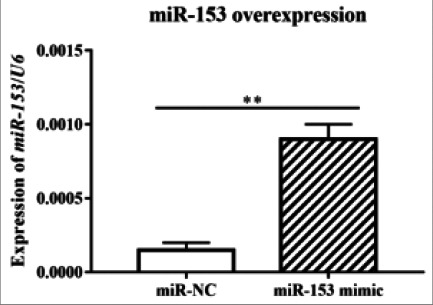
MiR-153 mimic transfection resulted in the overexpression of miR-153 in A549 cells. Note: In comparison with that in miR-NC group, the expression of miR-153 in miR-153 mimic group remarkably rose (**P<0.01)

**Figure 6:**
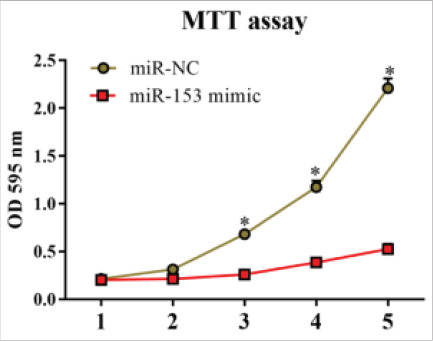
Overexpression of miR-153 inhibited the proliferation of A549 cells. Note: In contrast to that after transfection with miR-NC, the proliferative activity was markedly reduced on the 3rd, 4th and 5th day after transfection with miR-153 mimic (*P<0.05)

**Figure 7:**
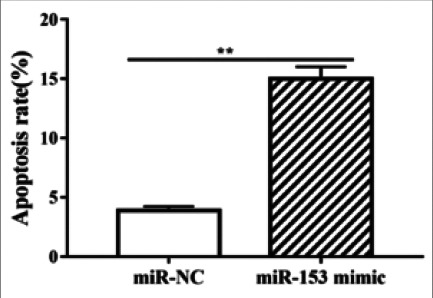
Overexpression of miR-153 facilitated the apoptosis of A549 cells. Statistics of the number of apoptotic cells. Note: In contrast to that in miR-NC group, the number of apoptotic cells in miR-153 mimic group was obviously increased (*P<0.05)

The expression of miR-153 was examined via qRT-PCR after A549 cells were transfected with miR-153 mimic. The results demonstrated that the expression of miR-153 in miR-153 mimic group was distinctly higher than that in miR-NC group (P<0.01, Figure 5). After the overexpression of miR-153, the changes in the proliferation and apoptosis of A549 cells were measured via MTT and TUNEL assays. It was found that compared with miR-NC group, miR-153 mimic group exhibited an evidently reduced cell proliferation rate (P<0.05, Figure 6), and a significantly increased number of apoptotic cells (P<0.05, Figure 7).

### Verification of the targeted relationship between miR-153 and PRDM2 via dual-luciferase reporter gene assay

**Figure 8:**
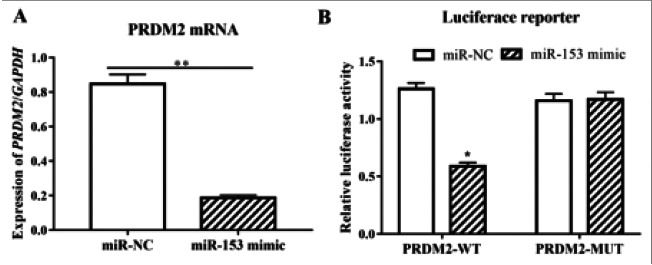
MiR-153 modulated PRDM2 expression in a targeted manner. (A) Compared with that in cells transfected with miR-NC, the mRNA level of PRDM2 prominently reduced in A549 cells (B) Transfected with miR-153 mimic following miR-153 overexpression, the transcriptional activity of PRDM2 in A549 cells was correspondingly downregulated Note: *P<0.05, **P<0.01

Firstly, the expression of PRDM2 was detected via qPCR after miR-153 overexpression, the results of which showed that compared with that in cells transfected with miRNC, the mRNA level of PRDM2 prominently reduced in A549 cells transfected with miR-153 mimic (P<0.01, Figure 8A). Then, to further confirm that PRDM2 is a downstream target gene of miR-153, dual-luciferase reporter assay system was employed for detection, and it was discovered that following miR-153 overexpression, the transcriptional activity of PRDM2 in A549 cells was correspondingly downregulated (P<0.05, Figure 8B), while the inhibitory effect of miR-153 disappeared after the mutation of PRDM2 3′-untranslated regions (UTRs).

### Changes in the levels of proteins in the JAK/STAT signaling pathway after miR-153 overexpression

**Figure 9:**
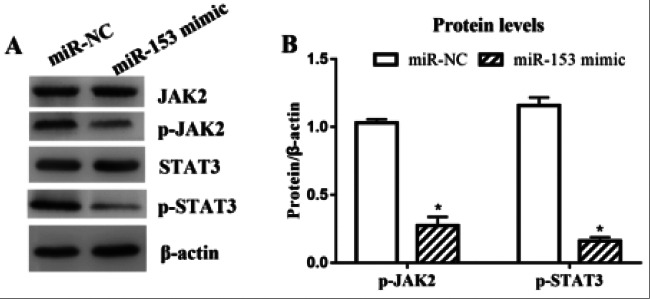
Protein levels of p-JAK2 and p-STAT3 were decreased after miR-153 overexpression. (A) Protein electrophoresis results. (B) Gray value analysis of protein bands. Note: *P<0.05

Western blotting assay results illustrated that compared with those in miR-NC group, the protein levels of p-JAK2 and p-STAT3 in miR-153 mimic group were significantly decreased (P<0.05, Figure 9), which suggests that the overexpression of miR-153 can effectively down-regulate the protein levels of p-JAK2 and p-STAT3.

## Discussion

In this study, the regulatory effects of miR-153/PRDM2 on the activity and apoptosis of A549 cells were investigated, and it was found that these processes were mediated by the JAK/STAT signaling pathway. The expression levels of miR-153 and PRDM2 were measured in NSCLC tissues and A549 cells, and it was observed that miR-153 expression was decreased while PRDM2 expression was increased in these samples. However, in A549 cells with miR-153 overexpression, PRDM2 expression was decreased, and a negative correlation was observed between miR-153 expression and PRDM2 expression. A dual-luciferase reporter gene assay was conducted to determine the direct regulatory relationship between miR-153 and the target gene PRDM2. Additionally, the activity and apoptosis induction of A549 cells following miR-153 overexpression were evaluated through MTT assay and TUNEL analysis, respectively. The results showed that the overexpression of miR-153 could suppress the activity of A549 cells and promote their apoptosis. This study is the first to demonstrate that miR-153 regulates the activity and apoptosis of NSCLC A549 cells by down-regulating PRDM2 and regulating the JAK/STAT signaling pathway.

According to a previous report, miRNAs are the most widely studied class of ncRNAs, which can trigger translation inhibition by identifying specific target mRNA sequences in the 3′-UTR in mammalian cells^38^. Therefore, miR-153 family can be used as a regulator of cell life and death based on specific cell environment and its targets. The differential expression of miR-153 in cancer cells changes tumor progression. Kim et al.,^39^ found that miR-153 is downregulated in ovarian epithelial tumors and is associated with the advanced clinical stage, which contributes to EMT and tumor metastasis of human epithelial cancer^40^. Up-regulation of miR-153 stimulates cell proliferation by down-regulating PTEN, a tumor suppressor gene, in human prostate cancer^41^. Moreover, the dysregulation of the JAK/STAT signaling pathway has been found in various diseases, and its activity is regulated by miRNAs^42^. For example, miR-155 is overexpressed in breast cancer and down-regulates the repressor protein SOCS1 to abnormally activate STAT3 signals^43^. JAK3 has been identified as a novel direct target of miR-221-3p in macrophages^44^. Although previous research has pointed out that miR-153 is differentially expressed in NSCLC tissues and exerts crucial effects in the development and progression of NSCLC, there has been no in-depth exploration of its mechanism. In this study, the downregulated miR-153 expression was detected in both NSCLC tissues and A549 cells. In A549 cells, the overexpression of miR-153 was indeed confirmed to inhibit cell proliferation and promote apoptosis, implying that the miR-153 overexpression exerts a protective effect in NSCLC tissues. In addition, Metascape was employed to analyze the function enrichment of the target genes of miR-153, so as to comprehensively research their functions and pathways. It was discovered that its target genes might participate in histone modification, transmembrane transport, membrane protein regulation and other processes. The above results provide a basis for further investigation of the action mechanism of miR-153.

The differential expression of miR-153 in NSCLC tissues has been reported for a long time, but no research has pointed out that miR-153 performs its functions by regulating the JAK/STAT signaling pathway. In this research, the overexpression of miR-153 was detected to inhibit the expression of its target gene PRDM2, inhibit the proliferation and promote the apoptosis of A549 cells by suppressing the activation of JAK2/STAT3 signaling pathway. Although the mechanism by which miR-153 triggers changes in the JAK2/STAT3 signaling pathway still needs further exploration, this research provides new insights for understanding the pathogenesis of NSCLC. Besides, this research not only improves the regulation mechanism of miR-153 in NSCLC, but also lays a foundation for further investigating the function of the JAK/STAT signaling pathway. The present study has several novel aspects. It is the first study to demonstrate that miR-153 regulates the activity and apoptosis of NSCLC cells by down-regulating PRDM2 and regulating the JAK/STAT signaling pathway. The study also provides new insights into the pathogenesis of NSCLC by improving the understanding of the regulation mechanism of miR-153 in NSCLC and laying a foundation for further investigation of the function of the JAK/STAT signaling pathway. Additionally, the study uses Metascape to analyze the function enrichment of the target genes of miR-153, which provides a basis for further investigation of the action mechanism of miR-153.

## Conclusions

The findings of this study have important clinical implications for the diagnosis and treatment of NSCLC. The differential expression of miR-153 in NSCLC tissues and cells was found to be related to the prognosis of NSCLC patients. Therefore, miR-153 could be used as a potential biomarker for the early diagnosis and prognosis of NSCLC. In addition, the overexpression of miR-153 was found to inhibit the proliferation and promote the apoptosis of A549 cells by suppressing the JAK2/STAT3 signaling pathway, suggesting that targeting miR-153 and the JAK/STAT pathway may have therapeutic potential for NSCLC treatment. These findings provide a promising direction for developing new therapeutic strategies for NSCLC patients. However, further research is needed to validate these findings in clinical trials and to explore the underlying molecular mechanisms of miR-153 in NSCLC. Overall, this study provides a foundation for further understanding the pathogenesis and treatment of NSCLC, and offers potential clinical benefits for NSCLC patients.

## Data Availability

The datasets used and analysed during the current study are available from the corresponding author on reasonable request.
